# Pregnane X Receptor Regulates Pathogen-Induced Inflammation and Host Defense against an Intracellular Bacterial Infection through Toll-like Receptor 4

**DOI:** 10.1038/srep31936

**Published:** 2016-08-23

**Authors:** Zhijuan Qiu, Jorge L. Cervantes, Basak B. Cicek, Subhajit Mukherjee, Madhukumar Venkatesh, Leigh A. Maher, Juan C. Salazar, Sridhar Mani, Kamal M. Khanna

**Affiliations:** 1 Department of Immunology, University of Connecticut Health Center, Farmington, CT 06030, USA; 2 Department of Pediatrics, University of Connecticut Health Center, Farmington, CT 06030, USA; 3 Departments of Genetics and Medicine, Albert Einstein College of Medicine, Bronx, NY10461, USA

## Abstract

The nuclear pregnane X receptor (PXR) plays a central role in regulating xenobiotic metabolism. We now report a novel role for PXR as a critical negative regulator of innate immunity after infection. *Pxr*^−/−^ mice exhibited remarkably elevated pro-inflammatory cytokine and chemokine production following infection with *Listeria monocytogenes* (*Lm*). Despite the more robust innate immune response, *Pxr*^−/−^ mice were highly susceptible to *Lm* infection. Surprisingly, disruption of the Toll-like receptor 4 (TLR4) but not TLR2 signaling restored the inflammation to normal levels and the ability to clear *Lm* in *Pxr*^−/−^ mice. Mechanistically, the heightened inflammation in *Pxr*^−/−^ mice resulted in the death of inflammatory monocytes that led to the enhanced susceptibility to *Lm* infection. These data demonstrated that PXR regulated pathogen-induced inflammation and host defense against *Lm* infection through modulating the TLR4 pathway. In summary, we discovered an apical role for PXR in regulating innate immunity. In addition, we uncovered a remarkable negative impact of the TLR4 pathway in controlling the quality of the inflammatory response and host defense against a gram-positive bacterial infection.

The pregnane X receptor (PXR), also known as steroid and xenobiotic receptor (SXR), pregnane activated receptor (PAR) or NR1l2, is a nuclear receptor that regulates the metabolism of xenobiotics by activating genes that are involved in xenobiotic absorption, distribution, metabolism and elimination^1^,^2^. We lately demonstrated that PXR activated by commensal bacterial metabolites also critically regulates gastrointestinal barrier function and integrity^3^.

Recently, PXR has been shown to regulate inflammation in intestine and liver^4^,^5^. A crosstalk between PXR and NF-κB signaling was suggested^4^,^6^. Activation of PXR by its ligand inhibits the expression of NF-κB target genes, while activation of NF-κB also suppresses PXR target gene expression. In both mouse and human studies there is evidence implicating a role of PXR in the pathogenesis of inflammatory bowel disease (IBD)^5^,^7^,^8^, which further reinforces the importance of PXR in regulating inflammation in the intestine and liver. Although PXR is shown to suppress NF-κB signaling pathway and thus inflammation in intestine and liver, majority of the studies have focused on the non-hematopoietic components such as hepatocytes and intestine epithelial cells. Since inflammation is a hallmark of innate immunity, a potential role of PXR in regulating innate immunity is worth exploring. Moreover, the role of PXR in the context of regulating pathogen-induced inflammation and antimicrobial immune response has not been investigated. We hypothesized that PXR would also inhibit NF-κB signaling in during innate immune responses, and that mice lacking PXR (*Pxr*^−/−^ mice) would exhibit elevated inflammation. Since inflammation is essential for bacterial clearance, we further hypothesized that *Pxr*^−/−^ mice would clear bacterial infection with greater efficiency. Indeed *Pxr*^−/−^ mice exhibited a strikingly elevated inflammatory response following *Listeria monocytogenes* (*Lm*) infection. However, surprisingly, *Pxr*^−/−^ mice were unable to clear *Lm* infection as efficiently as WT mice. We also showed that PXR expression by hematopoietic cells was important for the control of *Lm* infection. We further demonstrated that in the absence of PXR, the dysregulated inflammation was due to excessive activation of the TLR4 pathway. Blocking TLR4 but not TLR2 signaling in *Pxr*^−/−^ mice corrected the inflammation and rescued their ability to control *Lm* infection. We further demonstrated that the heightened inflammation in *Pxr*^−/−^ mice resulted in the death of inflammatory monocytes that led to enhanced susceptibility to *Lm* infection. Thus, our results demonstrate that PXR acts as a rheostat in fine tuning the balance of innate inflammatory pathways to ensure protective immunity.

## Results

### PXR is a negative regulator of innate inflammatory responses

**Figure 1:**
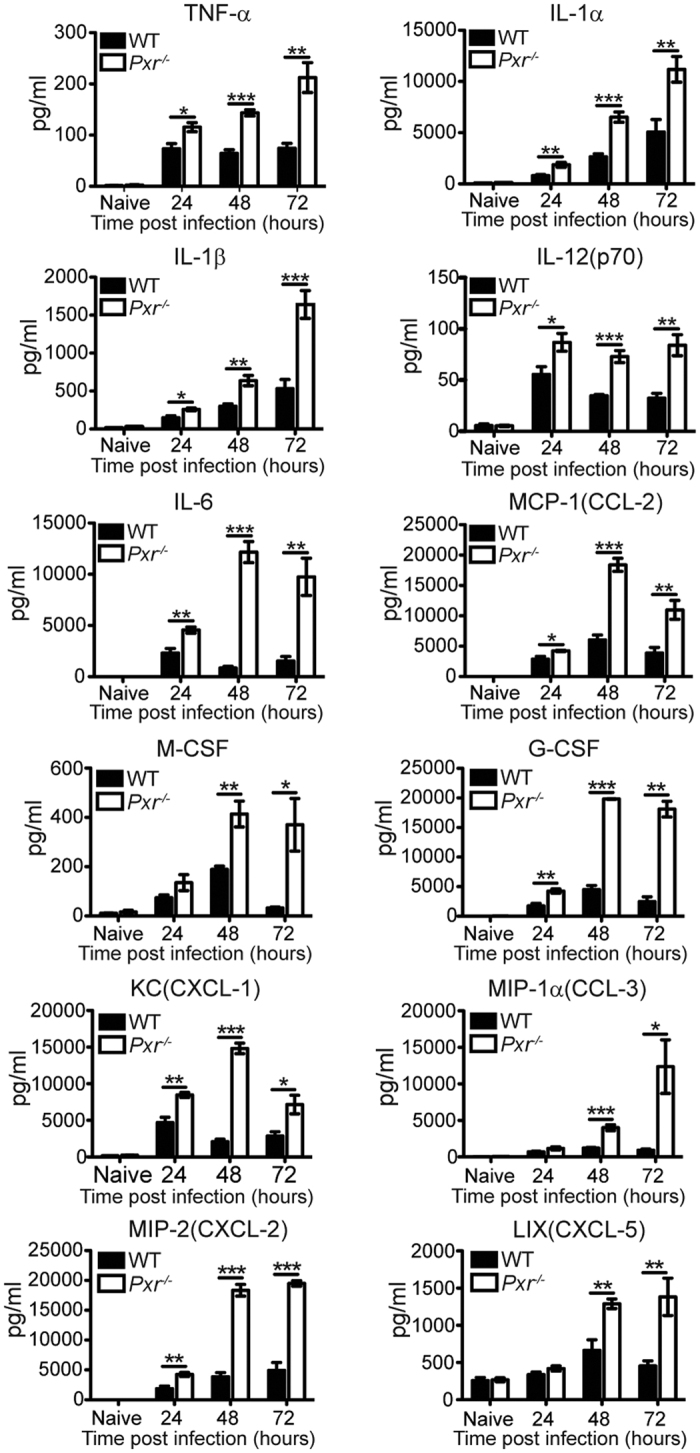
PXR is a negative regulator of innate inflammation. WT and *Pxr*^−/−^ mice were infected with *Lm.* Cytokine and chemokine productions in spleen at indicated time after infection were determined. n = 5. The data are presented as mean ± SEM and analyzed by two-tailed Student’s *t* test. *p < 0.05, **p < 0.01, and ***p < 0.005.

Since PXR was shown to suppress NF-κB signaling in intestinal epithelial cells and hepatocytes, we first wanted to determine whether PXR also regulated the inflammatory pathway during innate immune responses. *Lm* is a Gram-positive, facultative intracellular bacterium that induces potent innate inflammatory responses. We therefore assessed the production of pro-inflammatory cytokines in *Pxr*^−/−^ mice after *Lm* infection. There were no signs of overt inflammation in WT or *Pxr*^−/−^ mice during steady state (Fig. 1). However, after *Lm* infection in *Pxr*^−/−^ virtually every pro-inflammatory cytokine and chemokine detected was significantly higher when compared to WT mice (Fig. 1). These data demonstrated that indeed PXR functioned as a potent negative regulator of innate inflammatory responses.

### PXR is required for the control of Lm infection

**Figure 2:**
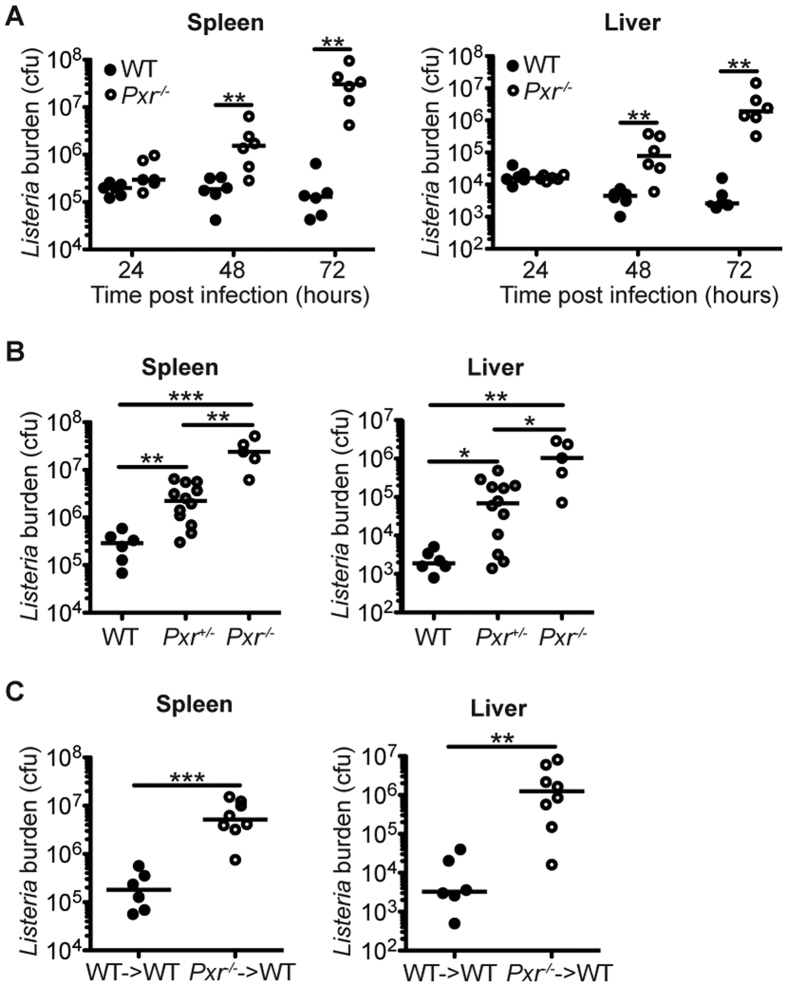
*Pxr*^−/−^ mice are highly susceptible to *Lm* infection. (**A**) WT and *Pxr*^−/−^ mice were infected with *Lm.* Bacterial burden was determined in spleen and liver at indicated time after infection. A representative of two independent experiments is shown. (**B**) WT, *Pxr*^+/−^ and *Pxr*^−/−^ mice were infected with *Lm* and bacterial burden was determined in spleen and liver at 72 hours after infection. A representative of two independent experiments is shown. (**C**) Bone marrow chimeras WT→WT or *Pxr*^−/−^→WT were infected with *Lm* and bacterial burden was determined at 72 hours after infection. A representative of two independent experiments is shown. The data were analyzed by Mann-Whitney test. Each dot represents one individual animal. Bar indicates median. *p < 0.05, **p < 0.01, and ***p < 0.005.

Innate inflammatory responses are critical for host defense against *Lm* infection^9^. Blocking the production of inflammatory cytokines such as TNF-α impairs the host defense against *Lm* infection^10^,^11^. Therefore, we wanted to further assess the physiological outcome of the increased inflammatory responses in *Pxr*^−/−^ mice. We expected that *Pxr*^−/−^ mice would clear *Lm* infection with greater efficiency. Surprisingly, in spite of the increased inflammatory response, *Pxr*^−/−^ mice were highly susceptible to systemic *Lm* infection. The bacterial load at 24 hours post infection were comparable in the spleen and liver of *Pxr*^−/−^ and WT mice (Fig. 2A). However, at 48 and 72 hours after infection the bacterial burden was significantly higher in *Pxr*^−/−^ mice in both spleen and liver (Fig. 2A). We further demonstrated that the ability of mice to clear *Lm* infection was dependent on *Pxr* gene copy number (Fig. 2B). Thus, these results showed that PXR served as a potent negative regulator of the innate inflammatory response, but paradoxically PXR was also required for the efficient control of *Lm* infection.

PXR is known to predominantly function in the stromal compartment such as intestinal epithelial cells and hepatocytes. We next determined whether PXR regulated host defense against *Lm* infection by a hematopoietic cell intrinsic mechanism. Lethally irradiated WT mice were reconstituted with bone marrow from WT or *Pxr*^−/−^ mice to generate WT→WT or *Pxr*^−/−^→WT chimeras respectively. Compared to WT→WT chimeras, *Pxr*^−/−^→WT chimeras failed to clear *Lm*, indicating that the expression of PXR in immune cells was important for the control of *Lm* infection (Fig. 2C).

### PXR regulates innate inflammation and host defense against Lm infection through TLR4

**Figure 3:**
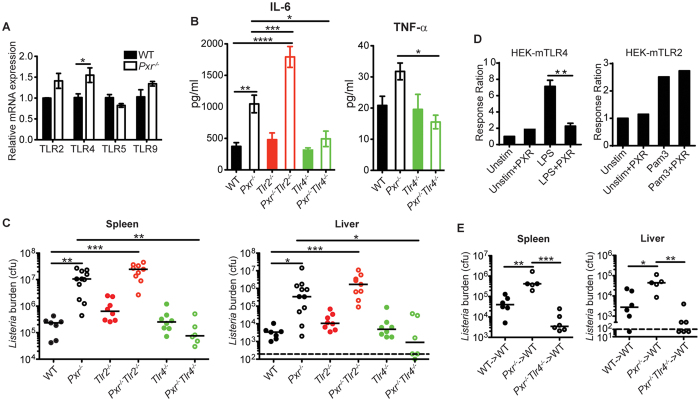
PXR regulates innate inflammation and host defense against *Lm* infection through TLR4. (**A**) TLR expression by splenocytes of naïve WT and *Pxr*^−/−^ mice. n = 5. The data are representative of 3 independent experiments. They are presented as mean ± SEM and analyzed by two-tailed Student’s *t* test. *p < 0.05. (**B**) IL-6 and TNF-α levels in serum of indicated mice at 24 hours after *Lm* infection were determined by multiplex bead based assay. n ≥ 5. A representative of 2 independent experiments is shown. The data are presented as mean ± SEM and analyzed by one-way ANOVA with Dunnett’s post-test to compare all other mice groups to WT mice group. *p < 0.05 and ***p < 0.005. (**C**) Bacterial burden in spleen and liver of indicated mice at 72 hours after *Lm* infection. A representative of 2 independent experiments is shown. The data were analyzed by Mann-Whitney test. Each dot represents one individual animal. Bar indicates median. *p < 0.05, **p < 0.01, and ***p < 0.005. (**D**) HEK-mTLR4 and HEK-mTLR2 cells were transfected with plasmid expressing PXR and stimulated with LPS or Pam3 respectively. The HEK-mTLR4 panel was pooled from three experiments and the HEK-mTLR2 panel was pooled from two experiments. They are presented as mean or mean ± SEM and analyzed by two-tailed Student’s *t* test. **p < 0.01. (**E**) Bacterial burden in spleen and liver of indicated mice at 72 hours after *Lm* infection. A representative of 2 independent experiments is shown. The data were analyzed by Mann-Whitney test. Each dot represents one individual animal. Bar indicates median. *p < 0.05, **p < 0.01, and ***p < 0.005.

Our results thus far demonstrated that in response to *Lm* infection, *Pxr*^−/−^ mice exhibited a more potent inflammatory response than WT mice. One explanation for the increased production of pro-inflammatory cytokines and chemokines after *Lm* infection in *Pxr*^−/−^ mice was enhanced TLR signaling. Therefore, we measured TLR mRNA levels in the spleen. We found that both TLR2 and TLR4 mRNA levels in splenocytes were increased in naive *Pxr*^−/−^ mice, with TLR4 showing the most significant upregulation (Fig. 3A). To determine whether the dysregulated inflammatory response in *Pxr*^−/−^ mice after *Lm* infection was due to enhanced TLR signaling, we crossed *Pxr*^−/−^ mice to *Tlr2*^−/−^ or *Tlr4*^−/−^ mice to generate *Pxr*^−/−^*Tlr2*^−/−^ and *Pxr*^−/−^*Tlr4*^−/−^ mice respectively. We then infected WT, *Pxr*^−/−^, *Tlr2*^−/−^, *Pxr*^−/−^*Tlr2*^−/−^, *Tlr4*^−/−^ and *Pxr*^−/−^*Tlr4*^−/−^ mice with *Lm* and assessed the production of pro-inflammatory cytokines at 24 hours. Our data showed that blocking TLR4 but not TLR2 signaling in *Pxr*^−/−^ mice restored the production of cytokines back to WT levels (Fig. 3B), suggesting that PXR negatively regulated the TLR4-induced inflammation.

Although the induction of pro-inflammatory pathways is requisite for host defense against bacterial infections, excessive inflammatory responses can be harmful to the host^12^. We postulated that the dysregulated inflammation was the main cause of the susceptibility of *Pxr*^−/−^ mice to *Lm* infection. Since contrary to *Pxr*^−/−^*Tlr2*^−/−^ mice, *Pxr*^−/−^*Tlr4*^−/−^ mice exhibited normal production of inflammatory cytokines, we hypothesized that *Pxr*^−/−^*Tlr4*^−/−^ mice would clear *Lm* infection efficiently while *Pxr*^−/−^*Tlr2*^−/−^ mice would not. To test this hypothesis, we determined bacterial burden in these mice at 72 hours after *Lm* infection. Indeed, *Pxr*^−/−^*Tlr4*^−/−^ mice were able to clear *Lm* as efficiently as WT mice (Fig. 3C). Conversely, *Pxr*^−/−^*Tlr2*^−/−^ mice failed to clear *Lm* and exhibited bacterial loads that were similar to those of *Pxr*^−/−^ mice (Fig. 3C). These data indicated that PXR regulated inflammation and host defense against *Lm* infection through modulating the TLR4 signaling pathway. To more directly demonstrate the differential regulation of TLR4 and TLR2 pathways by PXR, we performed *in vitro* experiments with HEK-mTLR4 and HEK-mTLR2 cells. The transfection with PXR significantly inhibited the response of HEK-mTLR4 cells to LPS but had no effect on the response of HEK-mTLR2 cells to Pam3 (Fig. 3D). Both the *in vivo* and *in vitro* experiments suggested that PXR specifically and negatively regulated the TLR4 pathway. We further demonstrated that the protective effect of TLR4 deficiency in *Pxr*^−/−^ mice was dependent on hematopoietic cells, since irradiated WT hosts transplanted with *Pxr*^−/−^*Tlr4*^−/−^ bone marrow cells were highly resistant to *Lm* infection (Fig. 3E), further confirming the hematopoietic cell intrinsic mechanism of regulation mediated by PXR.

### PXR regulates host defense against Lm infection by promoting inflammatory monocyte survival

**Figure 4:**
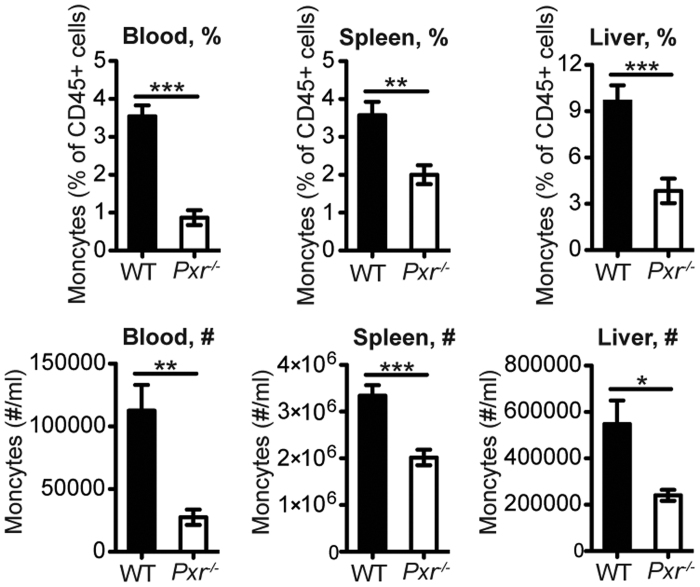
PXR deficiency results in the reduction of inflammatory monocytes. The percentage (%) and number (#) of inflammatory monocytes in blood, spleen and liver at 24 hours post infection. The data are representative of three independent experiments and presented as mean ± SEM and analyzed by two-tailed Student’s *t* test. *p < 0.05, **p < 0.01, and ***p < 0.005.

**Figure 5:**
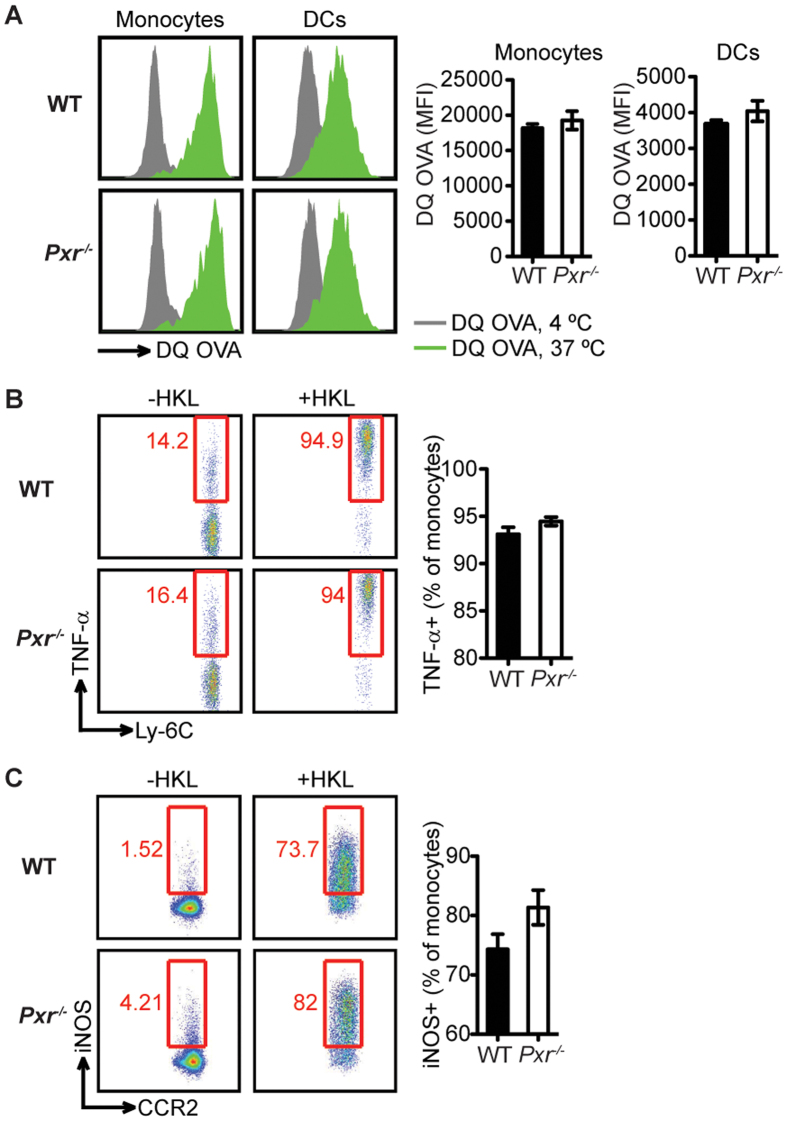
PXR deficiency does not impair the function of inflammatory monocytes. Mice were infected with *Lm* and data were collected at 24 hours later. The function of inflammatory monoyctes including the ability to phagocytose and degrade antigens (**A**) and the capacity to produce TNF-α (**B**) and iNOS (**C**) were measured. n = 3. A representative of three experiments is shown. The data are presented as mean ± SEM and analyzed by two-tailed Student’s *t* test.

**Figure 6:**
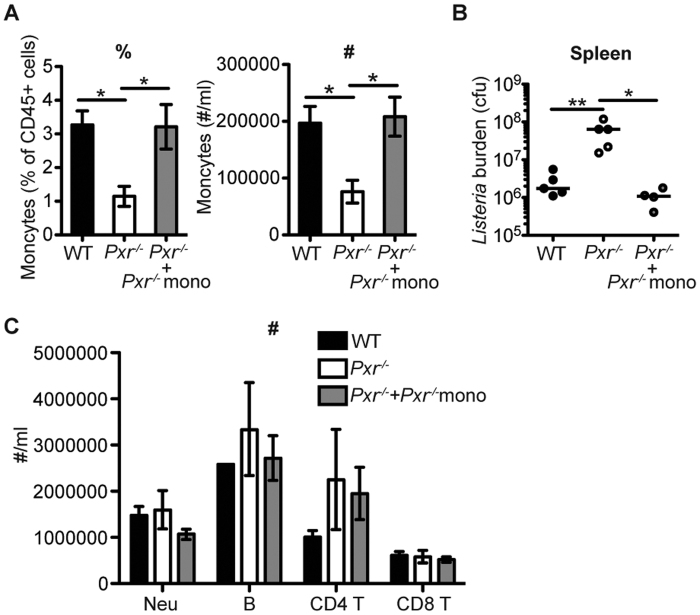
Adoptive transfer of inflammatory monocytes rescues the ability of *Pxr*^−/−^ mice to control *Lm* infection. Inflammatory monocytes were adoptively transferred into *Pxr*^−/−^ mice the day before infection. Data are representative of two independent experiments. (**A**) The percentage (%) and number (#) of inflammatory monocytes in the blood at 24 hours post infection. n ≥ 4. (**B**) Bacterial burden in the spleen of indicated mice at 72 hours post infection. (**C**) The number (#) of other immune subsets in the blood at 24 hours after infection were measured. n ≥ 4.

**Figure 7:**
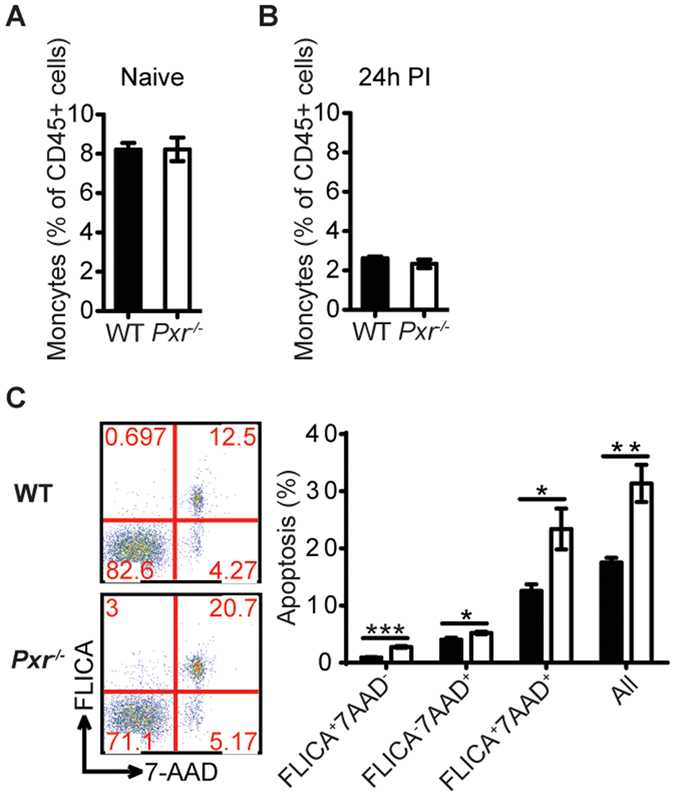
The reduction of inflammatory monocytes in *Pxr*^−/−^ mice is due to accelerated cell death. The frequency of inflammatory monocytes in the bone marrow of WT and *Pxr*^−/−^ mice in steady state (**A**) and at 24 hours post infection (**B**) was determined. n = 4. A representative of two experiments is shown. (**C**) Cell death of inflammatory monocytes in the spleen at 24 hours post infection was determined. n = 3. A representative of three experiments is shown. The data are presented as mean ± SEM and analyzed by two-tailed Student’s *t* test. *p < 0.05, **p < 0.01, and ***p < 0.005.

Next we attempted to determine the mechanism by which enhanced inflammatory responses in *Pxr*^−/−^ mice affected susceptibility to bacterial infection. We observed that both the frequency and number of inflammatory monocytes (identified as CD45^+^CD11b^+^Ly-6C^hi^CCR2^hi^) were markedly reduced in the blood, spleen and liver of *Pxr*^−/−^ mice at 24 hours post infection even when the bacterial burden was comparable between *Pxr*^−/−^ and WT mice (Fig. 4). Inflammatory monocytes produce significant amount of TNF-α and iNOS and are critical in clearing *Lm*^13^. However, we did not find any impairment in monocyte function, since they were as efficient as WT cells in phagocytosing and degrading particulate antigens and in producing TNF-α and iNOS (Fig. 5). We hypothesized that the reduction in the number of inflammatory monocytes was the leading cause of enhanced susceptibility of *Pxr*^−/−^ mice to *Lm* infection. Indeed, adoptive transfer of inflammatory monocytes (Fig. 6A) rescued the ability of *Pxr*^−/−^ mice to control *Lm* infection (Fig. 6B). This was not due to an effect on other types of immune cells as no change was observed in other immune cells after inflammatory monocyte transfer (Fig. 6C). The reduction in inflammatory monocytes was not due to their inability to migrate out of the bone marrow (Fig. 7A,B) but instead was due to accelerated monocytic cell death. Early apoptotic cells (FLICA^+^7AAD^−^), late apoptotic cells (FLICA^+^7AAD^+^) and necrotic cells (FLICA^−^7AAD^+^) were all significantly higher in *Pxr*^−/−^ mice at 24 hours post infection (Fig. 7C).

**Figure 8:**
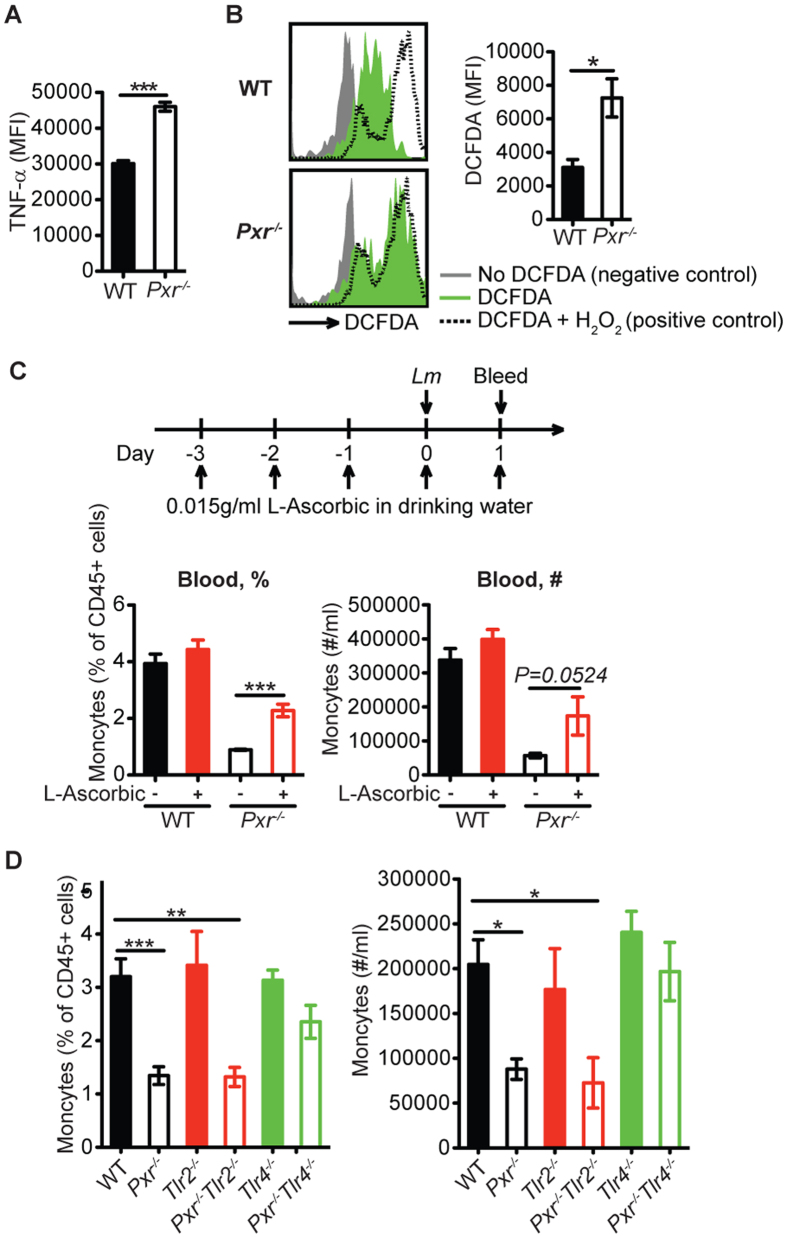
The excessive inflammation results in the increased inflammatory monocyte death in *Pxr*^−/−^ mice. (**A**) TNF-α production by inflammatory monocytes at per cell basis at 24 hours post infection. MFI, mean fluorescent intensity. n = 3. A representative of three experiments is shown. (**B**) ROS production by inflammatory monocytes at 24 hours post infection. n = 3. A representative of three experiments is shown. (**C**) Mice were treated with L-Ascorbic as indicated. The percentage (%) and number (#) of inflammatory monocytes in the blood at 24 hours post infection. n ≥ 5. (D) The percentage (%) and number (#) of inflammatory monocytes in the blood of indicated mice at 24 hours post infection. n ≥ 5. A representative of 2 independent experiments is shown. Only the comparisons of all other groups to WT group as well as the comparison of *Pxr*^−/−^*Tlr2*^−/−^ and *Pxr*^−/−^*Tlr4*^−/−^ groups to *Pxr*^−/−^ group are shown. The data are presented as mean ± SEM and analyzed by two-tailed Student’s *t* test. *p < 0.05, **p < 0.01, and ***p < 0.005.

Next we determined if excessive inflammation resulted in the death of inflammatory monocytes. TNF-α is known to contribute to cell death, and its downstream target reactive oxygen species (ROS) is also recognized to promote TNF-α-induced cell death^14^,^15^. Since inflammatory monocytes are a major source of TNF-α, we evaluated the magnitude of TNF-α production in monocytes after *Lm* infection. Although the percentage of monocytes that produced TNF-α was comparable between *Pxr*^−/−^ and WT mice (Fig. 5B), *Pxr*^−/−^ inflammatory monocytes produced significantly higher levels of TNF-α on a per-cell-basis as indicated by the mean fluorescent intensity (MFI) (Fig. 8A). We further observed that *Pxr*^−/−^ inflammatory monocytes produced higher ROS when compared to WT monocytes (Fig. 8B). In order to further tie the TNF-α - ROS pathway to cell death, we blocked ROS by treating *Pxr*^−/−^ mice with an antioxidant. We reasoned that the elevated ROS production was the major determinant of monocytic cell death in *Pxr*^−/−^ mice. Mice were treated with L-Ascorbic acid, an antioxidant and a ROS inhibitor. Antioxidant treatment rescued the inflammatory monocyte response in *Pxr*^−/−^ mice (Fig. 8C). These data suggested that the excessive ROS production was indeed responsible for the increased monocyte cell death in *Pxr*^−/−^ mice. We showed previously that blocking TLR4 but not TLR2 signaling in *Pxr*^−/−^ mice corrected the inflammation and rescued their ability to clear *Lm*. As expected, the inflammatory monocyte response was also restored in *Pxr*^−/−^*Tlr4*^−/−^ but not *Pxr*^−/−^*Tlr2*^−/−^ mice (Fig. 8D).

## Discussion

PXR is highly expressed in the intestine and liver where it functions as a master regulator of xenobiotic metabolism and gut barrier integrity^1^,^2^,^3^. More recently, its role in both endobiotic and xenobiotic metabolism has been tied into regulation of inflammation through NF-κB in intestine and liver stromal cells^4^,^5^,^6^,^7^,^8^. We have now identified a novel role of PXR as a critical hematopoietic cell intrinsic regulator of innate inflammation. These results establish that the mechanism by which PXR modulates the innate inflammatory response and host defense to *Lm* infection is through regulation of the TLR4 pathway. Thus, PXR has parallel functions: as a steroid and xenobitotic sensor and a critical regulator of innate immunity.

Our data indicated that TLR4 signaling was activated in *Pxr*^−/−^ mice after *Lm* infection, and the inflammation induced by TLR4 signaling was detrimental to host defense against the infection. However, *Lm* is a Gram-positive bacterium, and the prevailing concept is that TLR4 does not recognize any components of *Lm* and thus, is not activated during infection based on data generated from *Tlr4*^−/−^ mice and *in vitro* activation assays using TLR4 transfected human embryonic kidney (HEK) 293 cells^16^,^17^. However, it is possible that in *Pxr*^−/−^ mice, the threshold for the initiation of TLR4 signaling is considerably reduced due to the elevated TLR4 expression; consequently even those *Lm* components that are known to be ineffectual in inducing TLR4 signaling can in fact cause potent activation of the TLR4 pathway. It is also plausible that after *Lm* infection in *Pxr*^−/−^ mice the TLR4 pathway can be further fueled by endogenous ligands such as oxidized lipids^18^,^19^. Indeed we detected a significant increase in the production of ROS and iNOS. It is also conceivable that TLR4 pathway is in fact activated in WT mice after *Lm* infection, but the TLR4 signaling is antagonized by PX, and thus, the detrimental effect of TLR4 activation is not observed in the presence of PXR but is starkly evident when PXR is absent. These possibilities may not be mutually exclusive An important goal of future studies will be to dissect how PXR regulates TLR4 signaling, and determine the precise level at which PXR cross talks with the TLR signaling pathway. Overall, our results illustrate the need for exquisite fine tuning to balance innate inflammatory pathways to ensure the clearance of a pathogen. Our results have broad implications towards our understanding of how protective anti-microbial innate immune responses are regulated *in vivo,* and suggest a novel role of PXR as a critical negative regulator of the innate immune response after infection.

## Methods

### Mice and infections

Wild-type (WT) C57BL/6 mice were purchased from Charles River-National Cancer Institute (Frederick, MD). *Pxr*^−/−^ mice^20^ were generously provided by Dr. Sridhar Mani (Albert Einstein College of Medicine). *Tlr2*^−/−^ mice (B6.129-*Tlr2*^*tm1Kir*^/J) and *Tlr4*^−/−^ mice (B6.B10ScN-*Tlr4*^*lps-del*^/JthJ) were purchased from the Jackson Laboratory (Bar Harbor, ME). Bone marrow chimeras were generated by irradiation of recipient mice with 1000 rad followed by transplantation of 2 million donor bone marrow cells. They were allowed to reconstitute for 8 weeks before use. All procedures were carried out in accordance with National Institute of Health guidelines and were performed in accordance with and approved by the University of Connecticut Health Center Animal Care Committee.

The 10403s-derived recombinant *Listeria monocytogenes* strain that expresses a secreted form of OVA was used in the experiments. Infection was performed intravenously through tail vein at a dose of 1 × 10^4^ cfu. Infection doses were confirmed in every experiment by plating on BHI agar plates.

### Cytokine and Chemokine detection

Cytokines and chemokines in the serum or spleen were measured by using the Milliplex^TM^_MAP_ Mouse Cytokine/Chemokine Panels (Millipore). To collect serum, blood was allowed to clot at room temperature for 1 hour and at 4 °C for another hour before centrifugation at maximum speed for 15 minutes at 4 °C. To obtain spleen lysate, spleen was added to MagNA Lyser Green Beads (Roche) that contained 1 ml PBS followed by homogenization in MagNA Lyser Instrument (Roche) for 60 seconds at 6000 rpm. Following a quick spin at maximum speed for 1 min at 4 °C, the crude lysate was transferred to an Eppendorf tube and centrifuged at maximum speed for 15 minutes at 4 °C to further get rid of cell debris. The cytokine assay was performed by the CICATS Clinical Research Center Core Lab, at University of Connecticut Health Center as per manufacturer’s instructions.

### Bacteria burden

To determine bacteria burden, indicated tissues were harvested and homogenized in 1% saponin (Sigma-Aldrich). Homogenates were incubated at 4 °C for 1 hour to allow the release of intracellular bacteria. 10-fold serial dilutions were prepared and plated on BHI agar plates. Bacterial colonies were counted 48 hours after incubation at 37 °C.

### TLR expression analysis

Total RNA was extracted using RNeasy^®^ Mini Kit (Qiagen), and reverse transcribed into cDNA using iScript^TM^ cDNA Synthesis Kit (Bio-Rad). Quantitative real-time PCR was performed on CFX96TM Real-Time System (Bio-Rad) using TaqMan^®^ Gene Expression Assays (Applied Biosystems) including Mm00442346_m1 (*Tlr2*), Mm00445274_m1 (*Tlr4*), Mm00546288_s1 (*Tlr5*), Mm00446193_m1 (*Tlr9*), and Mm00607939_s1 (*β-actin*). Fold change 2^(−ΔΔCt)^ was used to determine the expression difference between WT and *Pxr*^−/−^ mice.

### *In vitro* assays with human embryonic Kidney (HEK) cells

HEK-Blue^TM^-mTLR2 and HEK-Blue^TM^-mTLR4 cells (InvivoGen) were transfected with 0.5 μg plasmid expressing mouse PXR. 24 hours later, cells were stimulated with 10 ng/ml LPS (Sigma-Aldrich) or Pam3CSK4 (Pam3, Sigma-Aldrich) for another 24 hours. The responses were measured and normalized to unstimulated cells.

### Flow cytometry

Inflammatory monocytes were identified as CD45^+^CD11b^+^Ly-6C^hi^CCR2^hi^ using antibodies for CD45 (Life Technologies), CD11b (BioLegend), Ly-6C (BioLegend) and CCR2 (R&D Systems). To determine the phagocytosis and degradation of antigens, splenocytes were labeled with DQ OVA (Life Technologies) and incubated at 37 °C for 1 hour, or at 4 °C in the presence of azide for 1 hour as negative control. To detect tumor necrosis factor (TNF)-α, reactive oxygen species (ROS) and inducible nitric oxide synthase (iNOS), splenocytes were stimulated with 1 × 10^8^ cfu/ml heat-killed *Lm* at 37 °C for 4 hours. TNF-α antibody and iNOS/NOS II polyclonal antibody were purchased from BD and Millipore respectively. Apoptosis assay was performed using CaspaTag™ Pan-Caspase *in situ* Assay Kit, Fluorescein (Millipore). ROS was measured using CM-H2DCFDA probe (DCFDA, Life Technologies) as previously described^21^. Hydrogen peroxide (H_2_O_2_, Sigma-Aldrich) was used as positive control.

### Inflammatory monocyte transfer

Inflammatory monocytes were enriched from spleens by depleting T cells, B cells, NK cells, DCs and neutrophils using PE-conjugated B220, CD3ε, CD4, CD8α, NK1.1, Ly-6G and CD11c antibodies (BioLegend) and Anti-PE MicroBeads (Miltenyi Biotec). Proximately 5 × 10^5^ inflammatory monocytes were transferred.

### Other reagents

L-Ascorbic acid was purchased from Sigma-Aldrich.

## Additional Information

**How to cite this article**: Qiu, Z. *et al*. Pregnane X Receptor Regulates Pathogen-Induced Inflammation and Host Defense against an Intracellular Bacterial Infection through Toll-like Receptor 4. *Sci. Rep.* **6**, 31936; doi: 10.1038/srep31936 (2016).
