# Corrigendum: Pregnane X Receptor Regulates Pathogen-Induced Inflammation and Host Defense against an Intracellular Bacterial Infection through Toll-like Receptor 4

**DOI:** 10.1038/srep44959

**Published:** 2017-03-22

**Authors:** Zhijuan Qiu, Jorge L. Cervantes, Basak B. Cicek, Subhajit Mukherjee, Madhukumar Venkatesh, Leigh A. Maher, Juan C. Salazar, Sridhar Mani, Kamal M. Khanna

Scientific Reports 6: Article number: 31936; 10.1038/srep31936 published online: 08 23 2016; updated: 03 22 2017.

The Acknowledgements section in this Article is incomplete.

“We thank Drs. Lynn Puddington, Anthony Vella and Leo Lefrançois for helpful discussions. This work was supported by NIH grant (AI097375) to KMK and funds provided by the UCONN Health”.

should read:

“We thank Drs. Lynn Puddington, Anthony Vella and Leo Lefrançois for helpful discussions. This work was supported by NIH grant (AI097375) to KMK and funds provided by the UCONN Health. This paper was also supported by the Broad Medical Research Program at CCFA (Crohn’s and Colitis Foundation of America) Investigator Award Proposal No. 362520 and NIH grants CA127231 and CA 161879 (to SM)”.

